# Publisher’s Note: *Journal of Health, Population and Nutrition*, volumes published in 2015

**DOI:** 10.1186/s41043-016-0046-0

**Published:** 2016-04-04

**Authors:** Journal of Health, Population and Nutrition

**Affiliations:** BioMed Central, Floor 6, 236 Gray’s Inn Road, London, WC1X 8HB UK

Due to a technical error in production, between June 2015 and December 2015, articles that should have been published in Volume 34 were published in Volume 33 giving the impression of a gap in volumes between Volume 33 (2015) and Volume 35 (2016). We apologise for any confusion caused to our readers.

